# Quality of life in Parkinson's disease: A systematic review and meta‐analysis of comparative studies

**DOI:** 10.1111/cns.13549

**Published:** 2020-12-28

**Authors:** Na Zhao, Yuan Yang, Ling Zhang, Qinge Zhang, Lloyd Balbuena, Gabor S. Ungvari, Yu‐Feng Zang, Yu‐Tao Xiang

**Affiliations:** ^1^ Unit of Psychiatry, Institute of Translational Medicine, Faculty of Health Sciences University of Macau Macao SAR China; ^2^ Center for Cognition and Brain Sciences University of Macau Macao SAR China; ^3^ Center for Cognition and Brain Disorders Institutes of Psychological Sciences Hangzhou Normal University Hangzhou China; ^4^ Institute of Advanced Studies in Humanities and Social Sciences University of Macau Macao SAR China; ^5^ The National Clinical Research Center for Mental Disorders & Beijing Key Laboratory of Mental Disorders Beijing Anding Hospital & The Advanced Innovation Center for Human Brain Protection School of Mental Health Capital Medical University Beijing China; ^6^ Department of Psychiatry University of Saskatchewan Saskatoon SK Canada; ^7^ Division of Psychiatry School of Medicine University of Western Australia/Graylands Hospital Perth WA Australia; ^8^ The University of Notre Dame Australia Fremantle WA Australia

**Keywords:** comparative study, meta‐analysis, Parkinson's disease, quality of life

## Abstract

**Background:**

Studies regarding the impact of Parkinson's disease (PD) on quality of life (QOL) have reported conflicting results, and the underlying QOL domains require further study. In order to understand the association between PD and QOL, we conducted this meta‐analysis to systematically compare QOL between PD patients and healthy controls.

**Method:**

The PubMed, PsycINFO, EMBASE, and Web of Science databases were systematically searched. Data were analyzed using the random‐effects model.

**Results:**

Twenty studies covering 2707 PD patients and 150,661 healthy controls were included in the study. Compared with healthy controls, PD patients had significantly poorer QOL overall and in most domains with moderate to large effects sizes. Different QOL measures varied in their association with quality of life, with the Parkinson's Disease Questionnaire‐39 (PDQ‐39) having the largest effect size (standard mean difference, SMD = −1.384, 95% CI: −1.607, −1.162, *Z* = 12.189, *P* < 0.001), followed by the Europe Quality of Life Questionnaire‐visual analogue scale (EQ‐VAS) (SMD = −1.081, 95% CI: −1.578, −0.584, *Z* = −4.265, *P* < 0.001), Europe Quality of Life Questionnaire‐5D (EQ‐5D) (SMD = −0.889, 95% CI: −1.181, −0.596, *Z* = −5.962, *P* < 0.001), and the Short‐form Health Survey (SF) scales (physical dimension: SMD = −0.826, 95% CI: −1.529, −0.123, *Z* = −2.303, *P* = 0.021; mental dimension: SMD = −0.376, 95% CI: −0.732, −0.019, *Z* = −2.064, *P* = 0.039).

**Conclusion:**

PD patients had lower QOL compared with healthy controls in most domains, especially in physical function and mental health. Considering the negative impact of poor QOL on daily life and functional outcomes, effective measures should be developed to improve QOL in this population.

## Graphical abstract

Improving the QOL in patients with PD is an important concern, which can provide reference for decisions of policymakers and clinicians. PD patients had significantly poorer QOL, with moderate to large effect sizes in most domains. Moreover, different QOL measures had moderating effects on the results.

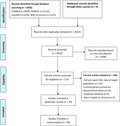

## 1 INTRODUCTION

Parkinson's disease (PD) is a neurodegenerative disease having an overall prevalence ranging from 1 to 2 per 1000 people.^1^, ^2^ PD is a chronic, progressive, age‐related disorder, which is rare in young people, but whose prevalence reaches up to 4% in older adults.^2^ PD is characterized by various motor dysfunctions, such as bradykinesia, rigidity, gait freezing, resting tremor, and postural reflex impairment,^3^ as well as neuropsychological dysfunctions, such as depression, fatigue, cognitive decline, and sleep disturbance,^4^ all of which negatively affect patients' quality of life (QOL).

The World Health Organization (WHO) defined QOL as “an individual's perception of their position in life in the context of the culture and value systems in which they live and in relation to their goals, expectations, standards, and concerns.”^5^ QOL encompasses physical, psychological, autonomy, cognitive, social relations, and environmental factors.^5^, ^6^ To improve the QOL of PD patients, it is important to understand how various QOL domains differ in PD patients and healthy controls. Some comparative studies on QOL in PD patients have been conducted, but the findings are mixed, especially the extent of differences between PD patients and controls in different domains. For instance, compared with healthy controls, some studies found that PD patients had an overall lower QOL,^7^, ^8^, ^9^, ^10^, ^11^, ^12^ while other studies did not find significant differences in QOL domains of physical health,^8^, ^13^ mental health,^9^ emotional function,^10^ environment,^11^ and social relations.^12^ Major correlates of QOL in PD include comorbid depressive symptoms, and PD severity and subtypes.^14^ Gait impairments, adverse effects of medications, and psychosocial dysfunction are contributing factors to poor QOL.^15^ To the best of our knowledge, no systematic review or meta‐analysis has compared QOL between PD patients and healthy controls that also drilled into various domains. The main objectives in this systematic review and meta‐analysis were as follows: (a) to compare the overall and domain QOL between PD patients and healthy controls and (b) to quantify QOL differences between groups, with different standardized instruments, using the effect size statistic. We hypothesized that PD patients would have significantly lower QOL compared with healthy controls.

## 2 METHODS

### 2.1 Search strategy

Two researchers (NZ and YY) independently and systematically searched the PubMed, PsycINFO, EMBASE, and Web of Science databases from their inception date until September 19, 2020, using the following search items: Parkinson disease, Parkinson's disease, life quality, health‐related quality of life, health‐related quality of life, HRQOL, case‐control, survey, cross‐sectional, and cohort. The references of relevant review articles were also searched manually for additional studies.

### 2.2 Inclusion and exclusion criteria

The search for relevant articles was conducted according to the Preferred Reporting Items for Systematic Reviews and Meta‐Analyses (PRISMA) flowchart,^16^ with the registration number CRD42020171092. The inclusion criteria are summarized by the PICOS acronym: (a) ***P***articipants: patients with PD according to study‐defined diagnostic criteria, such as the UK PD Society Brain Bank criteria^17^, ^18^ and the Movement Disorder Society (MDS) clinical diagnostic criteria for PD^19^; (b) ***I***ntervention: not applicable. (c) ***C***omparison: healthy controls; (d) ***O***utcomes: QOL measured by standardized instruments, such as the World Health Organization Quality of Life Questionnaire (WHOQOL), Parkinson's Disease Questionnaire‐39 (PDQ‐39), and the Short‐Form Health Survey (SF); (e) ***S***tudy design: comparative studies, such as case‐control and cohort studies (only the baseline data was extracted) published in English. Studies with meta‐analyzable data, ie, QOL means and standard deviations (*SD*), in PD patients and healthy controls were included for analyses. Studies conducted in special populations (eg, veterans) were excluded. The same two researchers (NZ and YY) screened the titles and abstracts of relevant literature and then read the full text to further assess eligibility. Any disagreement was discussed by the two above researchers, and if a consensus could not be reached, guidance was sought from a senior researcher (YTX).

### 2.3 Data extraction and quality assessment

Participant and study information, such as first author, publication year, sampling method, QOL measures, number of PD patients and controls, illness duration, and QOL scores, was extracted. For studies reporting QOL by a patient subgroup (eg, by gender), overall QOL was calculated by combining the QOL subgroup scores using a formula.^20^ Study quality was independently assessed by the same two researchers (NZ and YY) using the Newcastle‐Ottawa Scale (NOS) in three domains: selection, comparability and exposure.^21^, ^22^ The NOS total score was calculated by summing up all item scores.

### 2.4 Statistical analysis

Data were analyzed with the Comprehensive Meta‐analysis software, version 2.0 (CMA; https://www.meta‐analysis.com/). Data were combined across studies using the same QOL measure, which varied from one study to another. Physical and mental/psychological domains were measured separately with the WHOQOL and SF scales; thus, domain scores were pooled for each scale. For studies without SDs for QOL data, the SDs of other studies were averaged as previously done.^23^ Standardized mean differences (SMDs) in QOL between PD patients and healthy controls were calculated to estimate effect size. As a guide, SMDs of 0.2, 0.5, and 0.8 were considered small, moderate, and large effect sizes, respectively.^24^ Taking into account differences in sampling methods, study characteristics, and assessment tools, random‐effects models were used to synthesize data.^25^ Heterogeneity was assessed with Q and I square statistics. An I^2^ value of 50 percent or more^20^ indicated significant heterogeneity in which case possible sources of heterogeneity between subgroups were explored based on: (a) QOL measures (WHOQOL vs. SF scales vs. PDQ‐39 vs. Europe Quality of Life Questionnaire‐5D (EQ‐5D) vs. Europe Quality of Life Questionnaire‐visual analogue scale (EQ‐VAS)) and (b) QOL domains (physical health vs. mental/psychological health). Each subgroup was required to consist of at least 3 studies. If there were 10 or more studies, funnel plots were created and Egger's Rank test was conducted to assess possible publication bias.^26^ The significance level for meta‐analytic outcomes was set at 0.05 with two‐tailed tests.

## 3 RESULTS

### 3.1 Literature selection

**Figure 1:**
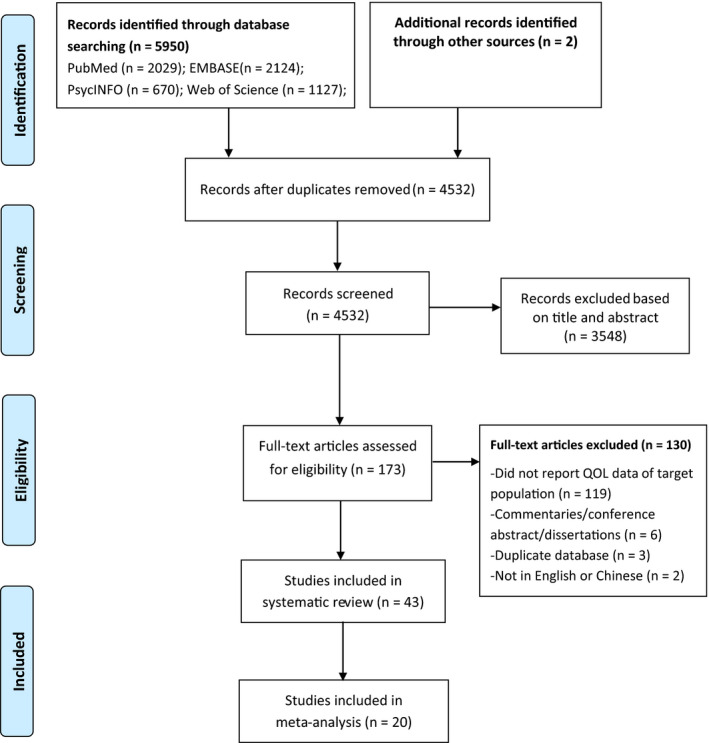
PRISMA flowchart

Figure 1 shows the result of the literature search. In total, 5950 studies were identified in target databases and 2 other studies were retrieved from reference lists. The final sample included in the meta‐analysis consisted of 20 studies with 2707 PD patients and 150,661 healthy controls.^8^, ^41^

### 3.2 Study characteristics and quality assessment

Key characteristics of included studies are summarized in Table 1. They were published between 1995 and 2020, and the sample size ranged from 33 to 144,692. The details of study quality assessment are presented in Table S1.

**Table 1:** Characteristics of studies included in this systematic review

	First author	References	Study site (Country)	Assessment of QOL	*N* total	*N* PD	*N* Controls	PD patients			Controls		NOS
								Age (Mean ± *SD*)	Male (%)	Disease duration, year (Mean ± *SD*)	Age (Mean ± *SD*)	Male (%)	
1	Adewusi et al, 2018	^27^	UK	SF‐36	104	52	52	68.1 ± 8.4	73.1	8.6 ± 5.9	66.8 ± 10.0	73.1	6
2	Arun et al, 2011	^28^	India	WHOQOL‐BREF	76	46	30	65.5 ± 9.4	67.4	4.3 ± 3.5	62.4 ± 8.4	70.0	6
3	Baig et al, 2015	^48^	UK	EQ‐5D	1056	769	287	67.7 ± 9.5	66.1	2.9 ± 1.9	65.3 ± 10.0	47.7	6
4	Barber et al, 2017	^49^	UK	EQ‐5D	415	119	296	66.9 ± 9.1	70.6	/	64.9 ± 10.2	49.0	7
5	Benli et al, 2016	^61^	Turkey	IPSS	79	39	40	69.8 ± 7.4	74.4	5.4 ± 3.5	68.0 ± 7.7	67.5	6
6	Chotinaiwattarakul et al, 2011	^29^	USA	SF‐36	226	134	92	70.7 ± 10.0	65.7	/	64.5 ± 9.9	27.2	5
7	Chu and Tan, 2018	^30^	Malaysia	PDQ‐39	109	54	55	66.8 ± 7.4	45.0	/	65.3 ± 7.5	51.0	6
8	Dogan et al, 2015	^55^	Turkey	PDQ‐39	171	86	85	64.3 ± 11.4	53.5	/	63.5 ± 10.7	51.8	7
9	Fan et al, 2018	^31^	UK	EQ‐5D/EQ‐VAS	1650	1050	600	62.6 ± 7.5	/	/	59.7 ± 7.2	/	5
10	Fonseca et al, 2015	^80^	Brazil	QOL‐AD	58	31	27	68.8 ± 10.4	67.7	/	74.0 ± 6.5	37.0	6
11	Greene and Camicioli, 2007	^32^	Canada	EQ‐5D	101	51	50	71.5 ± 4.7	58.8	8.7 ± 4.4	71.5 ± 4.8	58.0	7
12	Gustafsson et al, 2015	^60^	Sweden	LiSat‐11	2567	1432	1135	/	64.0	/	/	60.5	7
13	Haapaniemi et al, 2004	^56^	Finland	15D	2985	256	2729	/	/	/	/	/	5
14	Hariz and Forsgren, 2011	^10^	Sweden	SF‐36	130	99	31	69.0 ± 9.8	54.5	/	67.4 ± 6.6	54.8	6
15	Hendred and Foster, 2016	^11^	USA	WHOQOL‐BREF	156	96	60	62.4 ± 5.3	55.2	5.0 ± 4.3	61.7 ± 5.9	48.3	7
16	Hobson and Meara, 2018	^33^	UK	EQ‐5D	268	166	102	74.2 ± 8.6	73.5	13.2 ± 8.8	74.8 ± 6.6	59.8	5
17	Jakobsson et al, 2012	^34^	Sweden	SF‐12	3795	136	3659	70.5 ± 7.9	/	5.0 ± 4.9	85.7 ± 6.1	/	5
18	Jenkinson et al, 1995	^44^	UK	SF‐36	/	146	>=103	/	/	/	/	/	5
19	Kang et al, 2012	^35^	USA	SF‐36/PDQ‐39	33	15	18	65.7 ± 12.3	73.3	/	60.3 ± 13.5	50.0	6
20	Karlsen et al, 1999	^58^	Norway	NHP	333	233	100	73.6 ± 8.4	49.4	6.3 ± 5.3	72.8 ± 8.2	50.0	7
21	Kasten et al, 2012	^42^	Germany	WHOQOL‐BREF	255	128	127	63.0 ± 10.5	60.2	7.8 ± 6.3	59.0 ± 12.0	52.0	5
22	Larsen et al, 2000	^59^	Norway	NHP	261	161	100	/	/	/	/	/	5
23	Paolucci et al, 2018	^8^	Rome	SF‐36	396	29	367	66.1 ± 8.9	/	4.0 ± 2.1	/	/	6
24	Park et al, 2014	^13^	Korea	PDQ‐39	182	93	89	65.1 ± 9.8	41.9	3.5 ± 3.1	70.1 ± 6.0	51.1	6
25	Pohar and Jones, 2009	^57^	Canada	HUI3	111,968	261	111,707	68.9 ± 19.0	55.9	7.3 ± 13.6	44.8 ± 8.5	49.0	5
26	Prasuhn et al, 2017	^12^	Germany	WHOQOL‐BREF	327	69	258	68.0 ± 9.6	60.9	/	63.7 ± 7.1	48.4	6
27	Quittenbaum and Grahn, 2004	^45^	Sweden	SF‐36	152	57	95	70.1 ± 8.8	64.9	/	70.1 ± 8.3	69.5	7
28	Reuther et al, 2007	^50^	Germany	EQ‐5D/EQ‐VAS/PDQ‐39/PDQL	/	145	/	67.3 ± 9.6	66.9	9.3 ± 7.4	/	/	5
29	Riazi et al, 2003	^46^	UK	SF‐36	2283	227	2056	/	60.0	/	/	45.0	5
30	Santos Garcia et al, 2019	^81^	Spain	PQ‐10 /EUROHIS‐QOL8	901	694	207	62.6 ± 8.9	60.3	5.5 ± 4.4	61.0 ± 8.3	49.5	7
31	Schrag et al, 2000	^51^	UK	EQ‐5D/EQ‐VAS/ PDQ‐39/SF‐36	/	97	/	73.0 ± 11.3	51.5	5.8 ± 4.9	/	/	6
32	Swinn et al, 2003	^38^	UK	EQ‐5D/EQ‐VAS	118	77	40	62.8 ± 10.8	66.2	12.3 ± 5.3	60.2 ± 11.1	67.5	6
33	Tamás et al, 2014	^52^	Hungary	EQ 5D/EQ‐VAS/ PDQ‐39	831	110	721	63.3 ± 11.3	63.6	8.2 ± 5.8	/	/	6
34	Valeikiene et al, 2008	^39^	Lithuania	WHOQOL‐100	120	54	66	69.5 ± 6.8	53.7	/	68.5 ± 6.7	51.5	6
35	Vela et al, 2016	^53^	Spain	EQ‐5D/EQ‐VAS	174	87	87	46.9 ± 9.1	60.9	/	45.6 ± 8.6	54.7	6
36	Vossius et al, 2009	^9^	Norway	SF‐6D	371	199	172	67.7 ± 9.1	60.8	/	67.5 ± 9.1	60.0	7
37	Vescovelli et al, 2019	^43^	Europe	A general QOL question	103	50	53	70.6 ± 7.5	70	/	69 ± 8.7	69.8	6
38	Winter et al, 2010	^40^	Russia	EQ‐5D/EQ‐VAS	200	100	100	68.9 ± 7.0	38.0	6.7 ± 5.1	68.9 ± 58.7	38	7
39	Winter et al, 2011	^54^	Italy	EQ‐5D/EQ‐VAS	/	70	/	65.0 ± 8.5	58.6	/	/	/	5
40	Yamabe et al, 2018	^47^	Japan	SF‐6D	144,692	133	144,559	61.4 ± 14.3	54.1	/	48.2 ± 15.3	51.6	5
41	Yoon et al, 2017	^41^	Korea	PDQ‐39	125	89	36	68.5 ± 7.9	52.8	2.84 ± 3.21	65.2 ± 10.8	/	7
42	Pusswald et al, 2019	^37^	Austria	SF‐36	61	41	20	61.6 ± 8.87	50	/	64.44 ± 5.48	32	7
43	Prell et al, 2020	^36^	German	A novel QOL questionnaire	116	77	39	68.3 ± 8.90	55.8	8.8 ± 7.4	65.2 ± 10.1	25.6	7

Abbreviations: 15D, The generic 15D questionnaire; EQ‐5D, Europe Quality of Life Questionnaire‐5D; EQ‐VAS, Europe Quality of Life Questionnaire‐visual analogue scale; HUI3, The Health Utilities Index Mark 3; IPSS, The last question of the International Prostate Symptom Score; LiSat‐11, the Life Satisfaction Questionnaire; *N*, number; NHP, Nottingham Health Profile; EUROHIS‐QOL8, an 8‐item version of the WHOQOL‐BREF; PDQ‐39, the Parkinson's Disease Questionnaire‐39; PDQL, Parkinson's Disease Quality of Life; PQ‐10, a scale of global perceived QOL, from 0 (worst) to 10 (best); QOL‐AD, Quality of Life‐Alzhimer's Disease; SF‐12, SF‐6D, The short versions of SF scale; SF‐36, Short‐Form Health Survey (SF); WHOQOL‐100, World Health Organization Quality of Life Questionnaire; WHOQOL‐BREF, The short version of WHOQOL‐100.

### 3.3 QOL measurements

QOL measures involved in this systematic review are shown in Table 1. Five studies used the WHOQOL or its short version (WHOQOL‐BREF),^11^, ^12^, ^28^, ^42^, ^43^ of which 3 studies with available data^12^, ^28^, ^39^ were included in the meta‐analysis. Thirteen studies used the SF‐36, or its brief versions, such as SF‐12 and SF‐6D^8^, ^9^, ^10^, ^47^; 7 studies with available data were included in the meta‐analysis. Another twelve studies used EQ‐5D or EQ‐VAS^11^, ^54^; 4 studies using EQ‐5D^31^, ^33^, ^38^, ^49^ and 5 studies using EQ‐VAS^31^, ^32^, ^33^, ^38^, ^40^ with available data were included in the meta‐analysis.

Four studies applied PDQ‐39^13^, ^30^, ^41^, ^55^ and all of them had available data and were included in the meta‐analysis. Other QOL measures were also used such as the generic 15D questionnaire (15D),^56^ the Health Utilities Index Mark 3 (HUI3),^57^ Nottingham Health Profile (NHP),^58^, ^59^ the Life Satisfaction Questionnaire (LiSat‐11),^60^ an item of the International Prostate Symptom Score (IPSS)^61^ and a newly developed QOL questionnaire.^36^

Eventually, 20 studies with available data in both patient and control groups^8^, ^9^, ^37^, ^38^, ^39^, ^40^, ^41^, ^49^, ^52^ were included in the meta‐analysis.

### 3.4 QOL comparisons by scale

**Figure 2:**
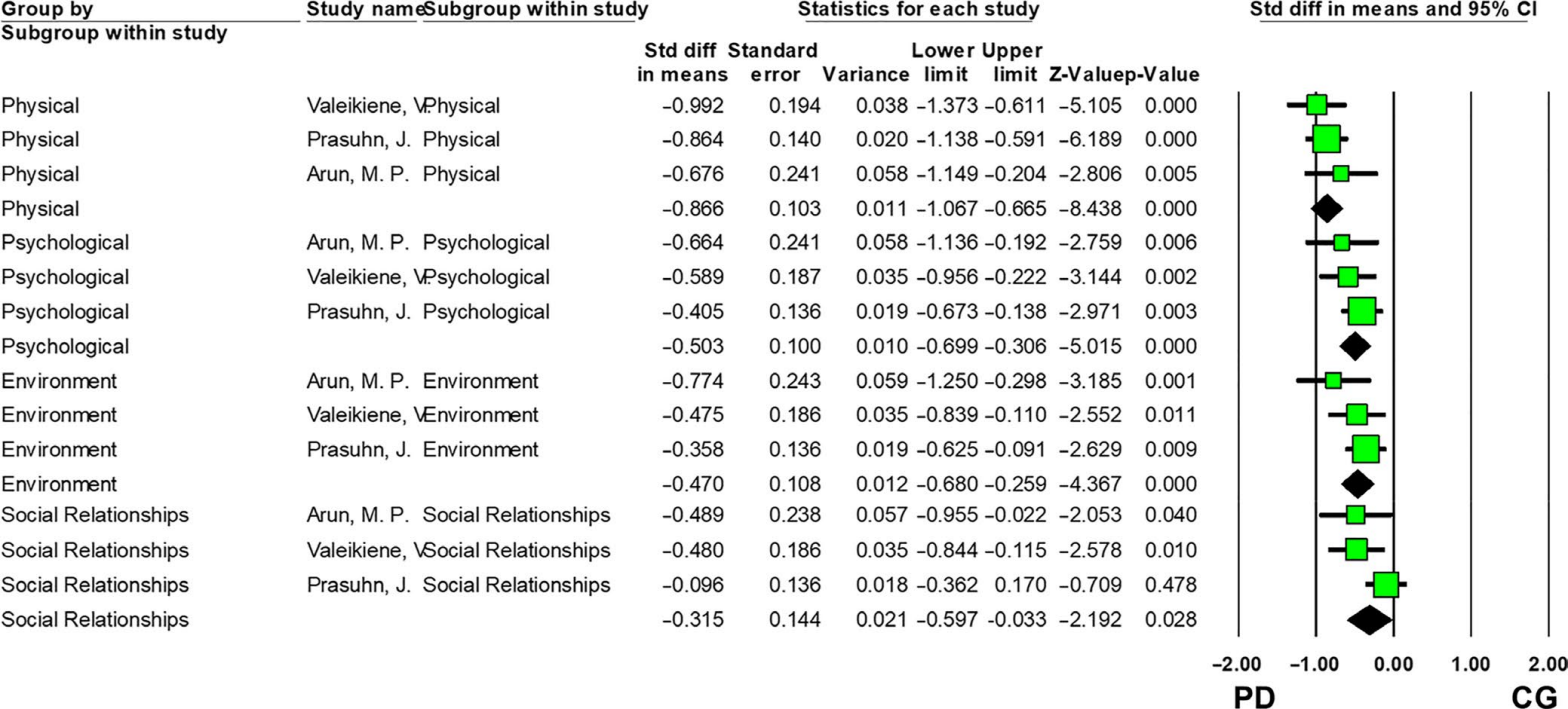
QOL comparison between PD patients and control group (CG) using WHOQOL

Three studies employing the WHOQOL^12^, ^28^, ^39^ were included in the meta‐analysis. Compared with healthy controls, PD patients had significantly poorer QOL in the physical domain with a large effect size (SMD = −0.866, 95% CI: ‐1.067, ‐0.665; *P* < 0.001), and the psychological (SMD = −0.405, 95% CI: 0.673, −0.138; *P* = 0.003), environmental (SMD = −0.470, 95% CI: −0.680, −0.259; *P* < 0.001), and social domains (SMD = −0.315, 95% CI: −0.597, −0.033; *P* = 0.028) with moderate effect sizes (Figure 2).

**Figure 3:**
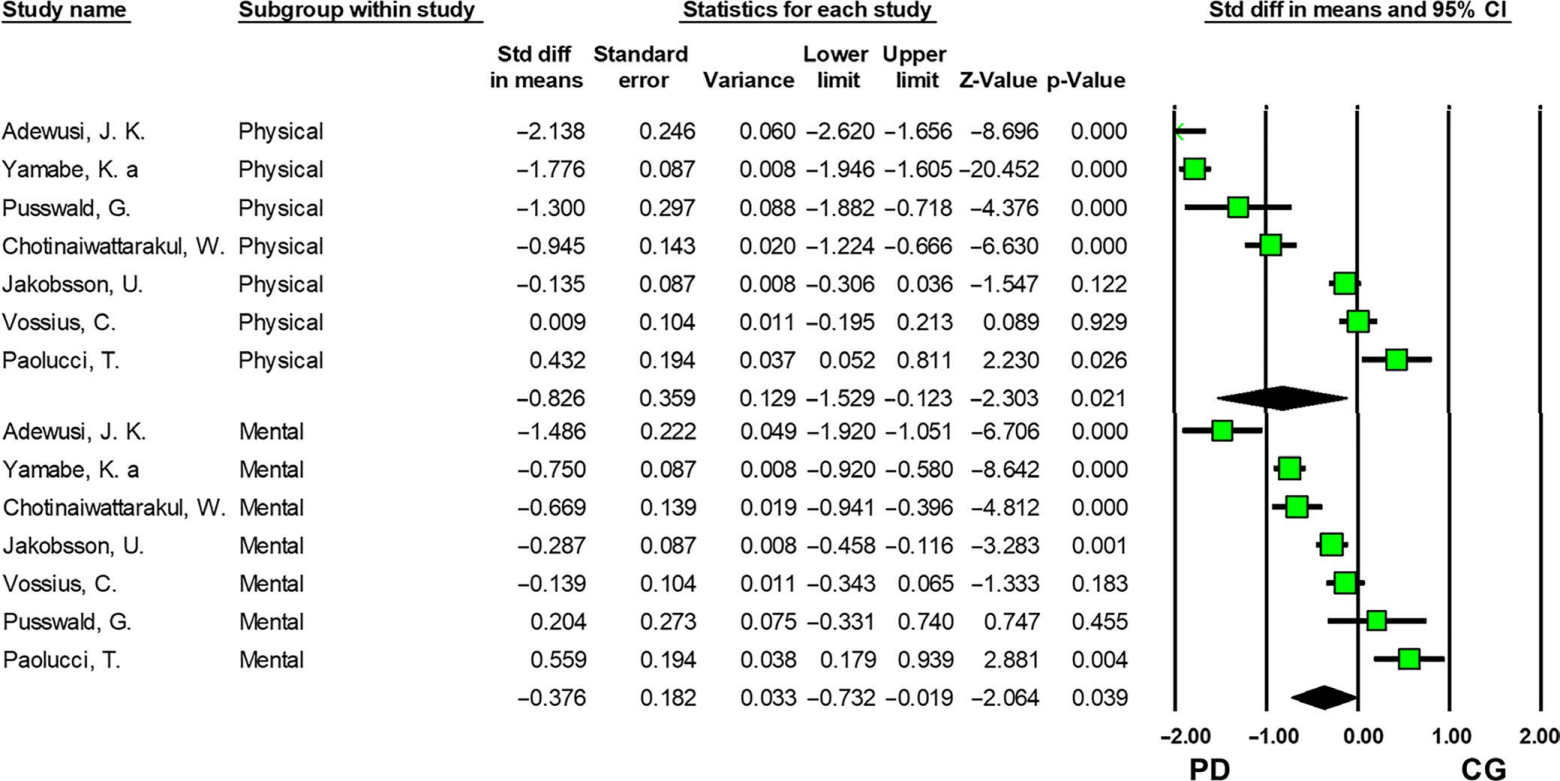
QOL comparisons between PD patients and control group (CG) using SF scales

Seven studies utilizing the SF scales were included in the meta‐analysis. Compared with healthy controls, the patient group had significantly poorer QOL in the physical domain with a large effect size (SMD = −0.826, 95% CI: −1.529, −0.123; P = 0.021), and in the mental domain with a moderate effect size (SMD = −0.376, 95% CI: −0.732, −0.019; P = 0.039) (Figure 3).

In order to increase statistical power, we pooled the studies with available data on physical and psychological/mental QOL domains in either the WHOQOL or SF scales. Compared with healthy controls, PD patients had significantly poorer QOL in the physical QOL with a large effect size (SMD = −0.857, 95% CI: −1.394, −0.321; *P* = 0.002), and in the psychological/mental QOL with a moderate effect size (SMD = −0.438, 95% CI: −0.726, −0.150; *P* = 0.003) (Figure S1).

Four studies using the PDQ‐39 (SMD = −1.384, 95% CI: −1.607, −1.162; Figure S2), 4 studies using the EQ‐5D) (SMD = −0.889, 95% CI = −1.181, −0.596, *P* < 0.001; Figure S3), and 5 studies applying the EQ‐VAS (SMD = −1.081, 95% CI = −1.578, −0.584, *P* < 0.001; Figure S4) were meta‐analyzed separately. Compared with controls, PD patients had significantly poorer overall QOL in these analyses.

### 3.5 Subgroup analyses and publication bias

No significant difference was found between the WHOQOL and SF assessments regarding physical and mental QOL (Table 2). There was a significant difference between QOL measures in effect sizes (Table 2); the PDQ‐39 was associated with the largest effect size, followed by the EQ‐VAS, EQ‐5D and SF scales (Table 2). Since the minimum number of studies per measure was not met, publication bias analysis could not be undertaken.

**Table 2:** Subgroup analyses of QOL between PD patients and healthy controls

Subgroups		Categories (number of studies)	Sample size		SMD	95% CI		I^2^	*P* within subgroup	Q (*P* value across subgropus)
			PD	HC		Lower	Upper			
Domain	Physical	WHOQOL (3)	169	354	−0.866	−1.067	−0.665	0	<0.001	1.351 (*P* = 0.245)
		SF (7)	724	148,921	−0.866	−1.593	−0.139	98.216	<0.001	
	Mental	WHOQOL (3)	169	354	−0.503	−0.699	−0.306	0	0.557	0 (*P* = 0.989)
		SF (7)	724	148,921	−0.598	−0.907	−0.289	89.961	<0.001	
Measurement of QOL	/	EQ‐5D (4)	1412	1098	−0.889	−1.181	−0.596	85.787	<0.001	188.353 (*P* < 0.001)
		EQ‐VAS (5)	1444	952	−1.081	−1.578	−0.584	94.362	<0.001	
		PDQ‐39 (4)	251	198	−1.384	−1.607	−1.162	7.645	0.355	
		SF(7)	724	148,921	−0.423	−1.131	0.285	98.194	<0.001	

## 4 DISCUSSION

To the best of our knowledge, this was the first systemic review and meta‐analysis that compared QOL between PD and healthy controls with standardized measures and estimating group differences. PD patients had significantly poorer QOL than healthy controls overall and in most domains.

Based on the distress/protection model of QOL, QOL is determined by the overall balance between protective and distressing factors.^62^ QOL is lower if distressing factors (eg, severe depressive symptoms) predominate over protective factors (eg, social support from family). Both motor and psychosocial dysfunctions and psychiatric comorbidities (eg, bradykinesia, rigidity, gait freezing, depression, fatigue, cognitive decline, and sleep disturbances associated with PD) are common in PD patients, which could lower their QOL. Certain demographic (eg, age,^11^, ^29^ gender,^39^ education level,^11^, ^63^ living condition,^43^, ^64^ knowledge and beliefs^64^ and marital status^40^) and clinical characteristics (eg, illness duration,^55^ and disease stage,^54^, ^55^, ^56^ severity^28^, ^42^, ^43^ and subtypes^10^, ^53^) were significantly associated with QOL in PD patients. The findings on the associations between psychiatric comorbidities and QOL in PD are conflicting. For example, depression was the strongest contributing factor for QOL in some,^11^, ^12^, ^28^ but not all studies.^55^ Anxiety, apathy, and pain are also associated with poor QOL in PD,^11^, ^48^ with greater effect sizes than motor‐symptoms.^48^ However, the significant relationship between anxiety and poor QOL was not found in another study.^12^ Similarly, the association between sleep disturbances and QOL is contested, with some studies finding a significant relationship between sleep problems and QOL,^7^, ^42^, ^58^ but not others.^12^, ^55^ In addition, some studies found that REM sleep behavior disorder with reduced striatal dopamine transporter values and increased expression of PD‐related pattern may be associated with the occurrence of PD.^65^, ^66^, ^67^ The discrepancy between studies could be partly due to differences in instruments,^68^, ^69^ sampling methods, disease severity,^14^, ^70^ effects of treatments,^71^, ^72^, ^73^, ^74^, ^75^ cognitive performance,^76^ and clinical presentations caused by different associated genes.^77^ The limited number of studies with the same QOL measure precluded an analysis of the moderating effects of the abovementioned demographic and clinical characteristics on QOL in PD.

Subgroup analyses revealed that QOL differences between PD patients and healthy controls varied by instrument (EQ‐5D vs. EQ‐VAS vs. PDQ‐39 vs. SF scales), probably resulting from the use of different items and emphasis between scales.^78^, ^79^ Two types of QOL measurements were applied, generic, and disease‐specific scales. Generic scales (eg, SF scales, EQ‐5D, and EQ‐VAS) are designed for all types of populations but may not be sensitive to PD‐related QOL. A disease‐specific scale (eg, PDQ‐39)^14^ is constructed for PD and detects minor differences in QOL. Hence, PD‐specific scales are clearly desirable clinical and research tools.

The strengths of this systematic review and meta‐analysis are the inclusion of comparative studies using standardized QOL measures and the large sample size (ie, 2707 PD patients and 150,661 healthy controls in the meta‐analysis) that improved statistical power and generalizability. However, several limitations should also be noted. First, different QOL measures were applied; therefore, in order to reduce heterogeneity attributable to measures, QOL was synthesized by QOL instrument. Second, some factors related to QOL, such as gender, illness duration, disease severity, health service system, and medication treatment, were not analyzed due to insufficient data in included studies. Third, causality between QOL and associated factors could not be explored due to the cross‐sectional design of the included studies. Fourth, only studies published in English were searched and limited number of studies conducted in developing countries were included.

In conclusion, PD patients had lower QOL compared with healthy controls in most dimensions, especially in physical function and mental health domains. Considering the negative impact of poor QOL on life and functional outcomes, factors contributing to poor QOL should be identified in longitudinal studies and effective measures should be developed to improve QOL in this population. For example, in order to improve QOL in physical function domain, physical rehabilitation together with the conventional pharmacotherapy and novel treatments, such as deep brain stimulation (DBS) surgery, could be considered. In contrast, timely adjunctive psychotherapy and psychotropic medications should be offered to appropriate PD patients in order to improve their mental health QOL.

## CONFLICT OF INTEREST

The authors have no conflicts of interest to declare.

## Supporting information

Supplementary Material

## Data Availability

Data sharing is not applicable to this article as no new data were created in this study.
