# Supplementary material for: Quality of life in Parkinson's disease: A systematic review and meta‐analysis of comparative studies
Source: CNS Neurosci Ther. 2020 Dec 28;27(3):270–9. doi: 10.1111/cns.13549 (PMC7871788; doi:10.1111/cns.13549)
Supplement: Supplementary file 1 — Supplementary Material [file CNS-27-270-s001.docx]

Supplementary Table 1. Study quality using the Newcastle-Ottawa Scale

| No. | First author, Year | Total Score | **Selection** | | | | **Comparability （Comparability of cases and controls on the basis of the design or analysis）** | | **Exposure** | | |
| --- | --- | --- | --- | --- | --- | --- | --- | --- | --- | --- | --- |
|  |  |  | 1) Is the patient case definition adequate (the inclusion criteria of PD group were clearly stated) | 2) Representativeness of the PD cases (consecutive or obviously representative *; potential bias or not stated) | 3) Selection of controls (community *; hospital controls; no description) | 4) Definition of controls (no history disease *; no description) | a) Study controls for age (Select the most important factor *.) | b) study controls for any additional factor *, e.g., educational level | 1) Ascertainment of exposure (secure record *; structured interview where blind to case/control status*; others) | 2) Same method of ascertainment for cases and controls (yes *; no) | 3) Non-Response rate (same rate for both groups *; no description) |
| 1 | Adewusi et al., 2018 | 6 | 1 | 1 | 1 | 1 | 1 | 0 | 0 | 1 | 0 |
| 2 | Arun et al., 2011 | 6 | 1 | 1 | 0 | 1 | 1 | 1 | 0 | 1 | 0 |
| 3 | Baig et al., 2015 | 6 | 1 | 1 | 1 | 1 | 1 | 0 | 0 | 1 | 0 |
| 4 | Barber et al., 2017 | 7 | 1 | 1 | 1 | 1 | 1 | 1 | 0 | 1 | 0 |
| 5 | Benli et al., 2016 | 6 | 1 | 0 | 1 | 1 | 1 | 1 | 0 | 1 | 0 |
| 6 | Chotinaiwattarakul et al., 2011 | 5 | 1 | 1 | 1 | 1 | 0 | 0 | 0 | 1 | 0 |
| 7 | Chu and Tan, 2018 | 6 | 1 | 1 | 1 | 1 | 1 | 0 | 0 | 1 | 0 |
| 8 | Dogan et al., 2015 | 7 | 1 | 1 | 1 | 1 | 1 | 1 | 0 | 1 | 0 |
| 9 | Fan et al., 2018 | 5 | 0 | 1 | 1 | 1 | 0 | 1 | 0 | 1 | 0 |
| 10 | Fonseca et al., 2015 | 6 | 1 | 1 | 1 | 1 | 0 | 1 | 0 | 1 | 0 |
| 11 | Greene and Camicioli, 2007 | 7 | 1 | 1 | 1 | 1 | 1 | 1 | 0 | 1 | 0 |
| 12 | Gustafsson et al., 2015 | 7 | 1 | 1 | 1 | 1 | 1 | 1 | 0 | 1 | 0 |
| 13 | Haapaniemi et al., 2004 | 5 | 1 | 1 | 1 | 0 | 1 | 0 | 0 | 1 | 0 |
| 14 | Hariz and Forsgren, 2011 | 6 | 1 | 1 | 1 | 1 | 1 | 0 | 0 | 1 | 0 |
| 15 | Hendred and Foster, 2016 | 7 | 1 | 1 | 1 | 1 | 1 | 1 | 0 | 1 | 0 |
| 16 | Hobson and Meara, 2018 | 5 | 1 | 1 | 0 | 1 | 1 | 0 | 0 | 1 | 0 |
| 17 | Jakobsson et al., 2012 | 5 | 1 | 1 | 1 | 1 | 0 | 0 | 0 | 1 | 0 |
| 18 | Jenkinson et al., 1995 | 5 | 1 | 1 | 0 | 1 | 1 | 0 | 0 | 1 | 0 |
| 19 | Kang et al., 2012 | 6 | 1 | 1 | 1 | 1 | 1 | 0 | 0 | 1 | 0 |
| 20 | Karlsen et al., 1999 | 7 | 1 | 1 | 1 | 1 | 1 | 1 | 0 | 1 | 0 |
| 21 | Kasten et al., 2012 | 5 | 1 | 1 | 1 | 1 | 0 | 0 | 0 | 1 | 0 |
| 22 | Larsen et al., 2000 | 5 | 1 | 1 | 1 | 1 | 0 | 0 | 0 | 1 | 0 |
| 23 | Paolucci et al., 2018 | 6 | 1 | 1 | 1 | 1 | 1 | 0 | 0 | 1 | 0 |
| 24 | Park et al., 2014 | 6 | 1 | 1 | 1 | 1 | 0 | 1 | 0 | 1 | 0 |
| 25 | Pohar and Jones, 2009 | 5 | 0 | 1 | 1 | 1 | 0 | 1 | 0 | 1 | 0 |
| 26 | Prasuhn et al., 2017 | 6 | 1 | 1 | 1 | 1 | 1 | 0 | 0 | 1 | 0 |
| 27 | Quittenbaum and Grahn, 2004 | 7 | 1 | 1 | 1 | 1 | 1 | 1 | 0 | 1 | 0 |
| 28 | Reuther et al., 2007 | 5 | 1 | 1 | 1 | 1 | 0 | 0 | 0 | 1 | 0 |
| 29 | Riazi et al., 2003 | 5 | 1 | 1 | 1 | 1 | 0 | 0 | 0 | 1 | 0 |
| 30 | Santos Garcia et al., 2019 | 7 | 1 | 1 | 1 | 1 | 1 | 1 | 0 | 1 | 0 |
| 31 | Schrag et al., 2000 | 6 | 1 | 1 | 1 | 1 | 1 | 0 | 0 | 1 | 0 |
| 32 | Swinn et al., 2003 | 6 | 1 | 1 | 1 | 1 | 1 | 0 | 0 | 1 | 0 |
| 33 | Tamás et al., 2014 | 6 | 1 | 1 | 1 | 0 | 1 | 1 | 0 | 1 | 0 |
| 34 | Valeikiene et al., 2008 | 6 | 1 | 1 | 1 | 1 | 1 | 0 | 0 | 1 | 0 |
| 35 | Vela et al., 2016 | 6 | 1 | 1 | 1 | 1 | 1 | 0 | 0 | 1 | 0 |
| 36 | Vossius et al., 2009 | 7 | 1 | 1 | 1 | 1 | 1 | 1 | 0 | 1 | 0 |
| 37 | Vescovelli et al., 2019 | 6 | 1 | 1 | 1 | 1 | 1 | 0 | 0 | 1 | 0 |
| 38 | Winter et al., 2010 | 7 | 1 | 1 | 1 | 1 | 1 | 1 | 0 | 1 | 0 |
| 39 | Winter et al., 2011 | 5 | 1 | 1 | 1 | 0 | 0 | 0 | 0 | 1 | 0 |
| 40 | Yamabe et al., 2018 | 5 | 1 | 1 | 1 | 0 | 1 | 0 | 0 | 1 | 0 |
| 41 | Yoon et al., 2017 | 7 | 1 | 1 | 1 | 1 | 1 | 0 | 0 | 1 | 0 |
| 42 | Pusswald et al., 2019 | 7 | 1 | 1 | 1 | 1 | 1 | 1 | 0 | 1 | 0 |
| 43 | Prell et al., 2020 | 7 | 1 | 1 | 1 | 1 | 1 | 1 | 0 | 1 | 0 |

**
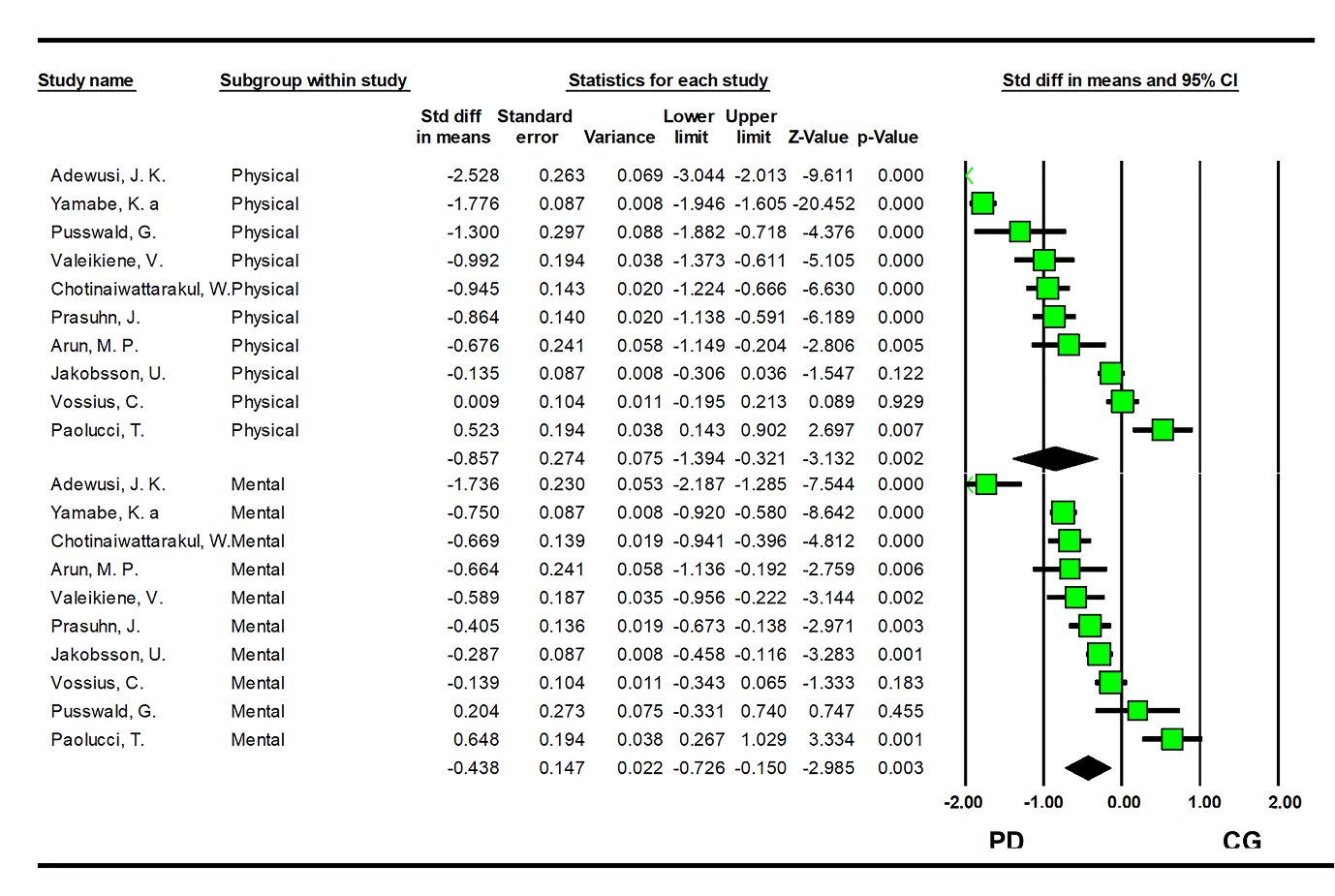
**

Supplementary Fig.1. QOL comparison between PD patients and healthy controls using WHOQOL or SF scales


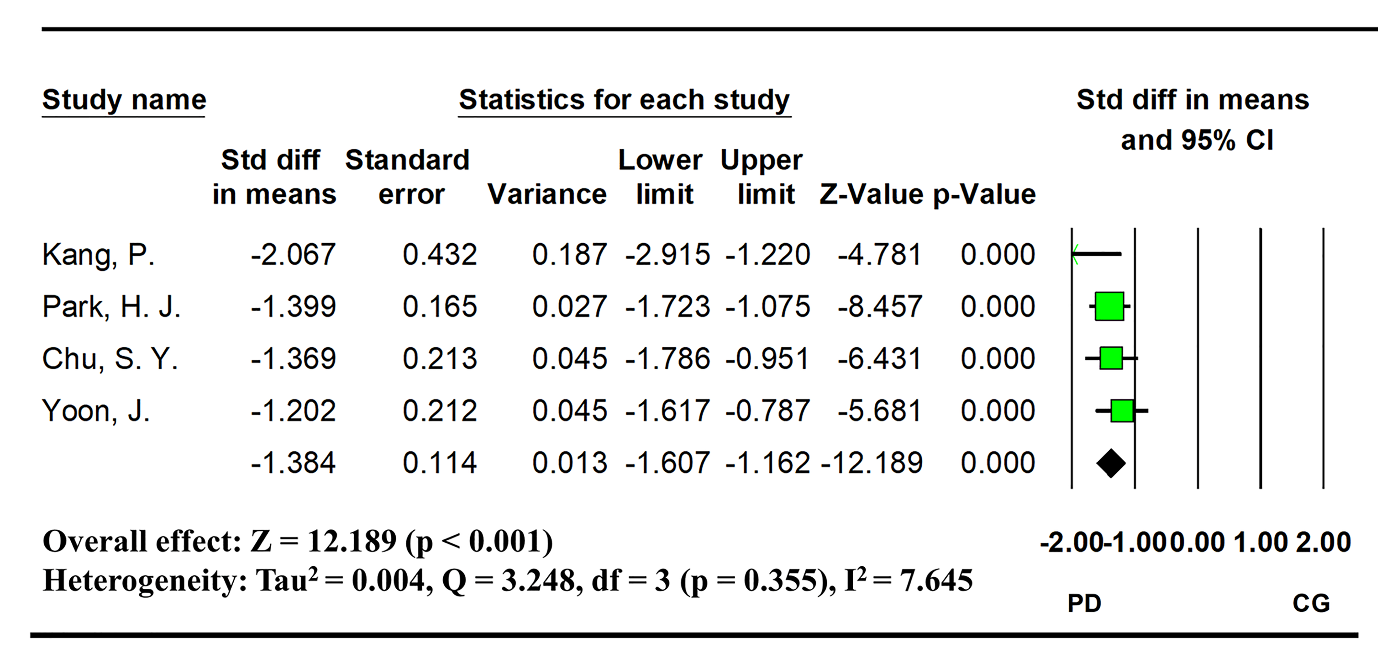


Supplementary Figure 2. QOL comparison between PD patients and healthy controls using PDQ-39


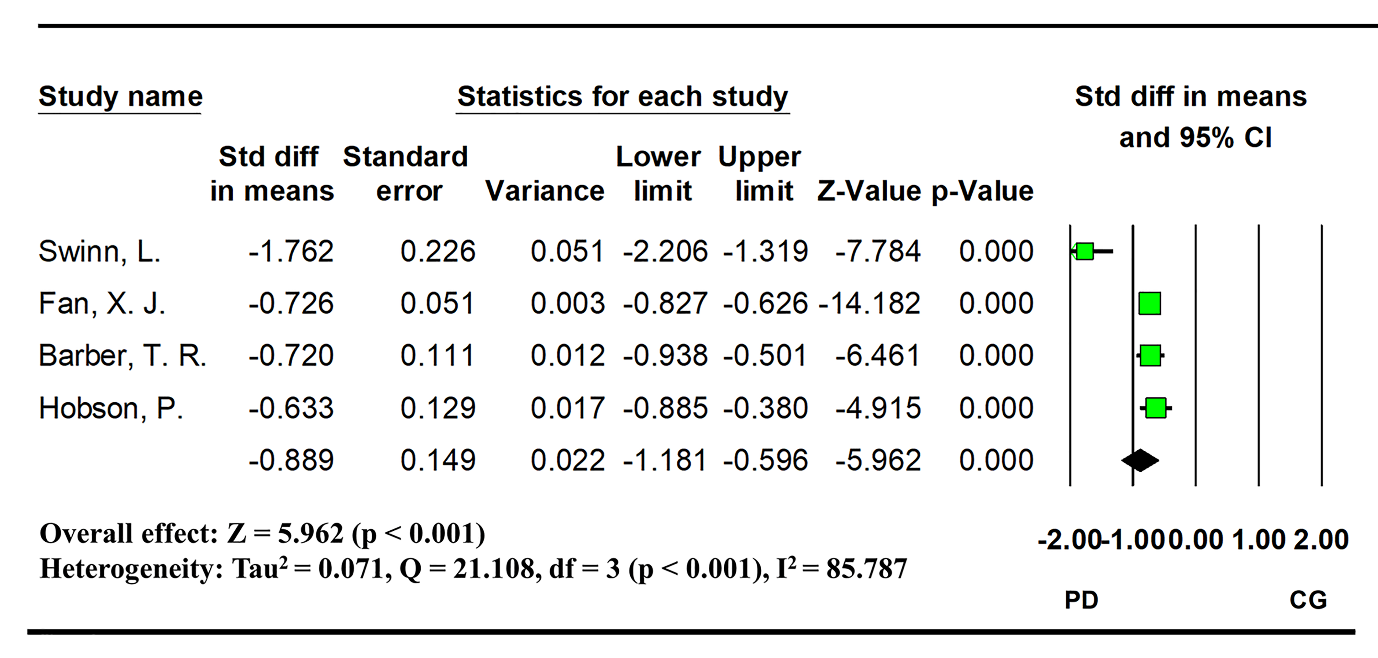


Supplementary Figure 3. QOL comparison between PD patients and healthy controls using EQ-5D


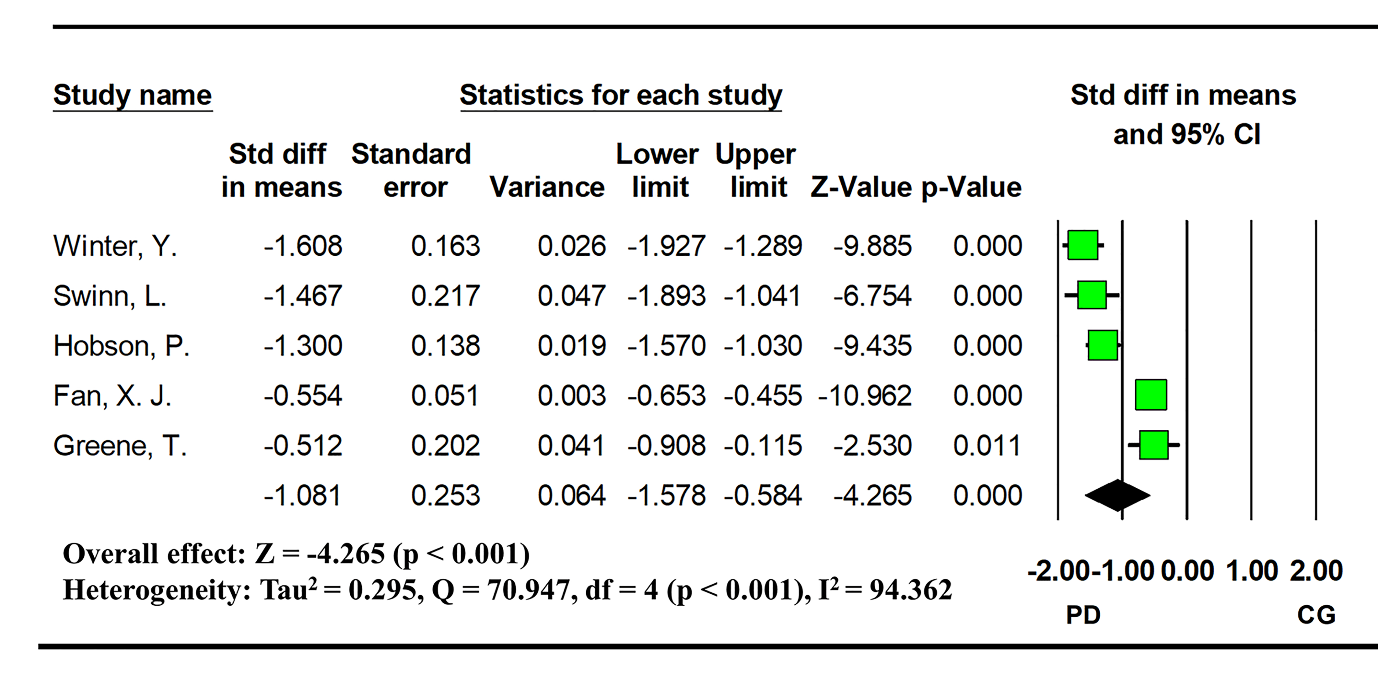


Supplementary Figure 4. QOL comparison between PD patients and healthy controls using EQ-VAS
